# Host Ant Change of a Socially Parasitic Butterfly (*Phengaris alcon*) through Host Nest Take-Over

**DOI:** 10.3390/insects11090556

**Published:** 2020-08-20

**Authors:** András Tartally, Anna Ágnes Somogyi, Tamás Révész, David R. Nash

**Affiliations:** 1 Department of Evolutionary Zoology and Human Biology, University of Debrecen, Egyetem tér 1, H-4032 Debrecen, Hungary; panka.somogyi@gmail.com (A.Á.S.); gigatamas@gmail.com (T.R.); 2 Juhász-Nagy Pál Doktoral School, University of Debrecen, Egyetem tér 1, H-4032 Debrecen, Hungary; 3 Centre for Social Evolution, Department of Biology, University of Copenhagen, Universitetsparken 15, DK-2100 Copenhagen, Denmark; DRNash@bio.ku.dk

**Keywords:** alcon blue, *Maculinea rebeli*, *Myrmica*, cuckoo strategy, adaptation, multi-host, mimicry

## Abstract

**Simple Summary:**

The endangered Alcon blue butterfly (*Phengaris alcon*) starts its larval stage by feeding on the seeds of gentian plants, after which it completes development in the nests of suitable *Myrmica* ant species. Any particular population often uses more than one host ant species, and some host switching is likely. To test switching in the lab we introduced relatively strong colonies of alien *Myrmica* species to the arenas of weaker colonies, and to orphaned caterpillars. Most of the caterpillars were successfully readopted by alien ants, and survived well. Our results suggest higher ecological plasticity in host ant usage of this butterfly than generally thought. The Alcon blue is an iconic species, e.g., its special life cycle has featured in several high profile television and streaming media wildlife series, and the more we know about its unusual life the more we can do for its protected sites.

**Abstract:**

The socially parasitic Alcon blue butterfly (*Phengaris alcon*) starts its larval stage by feeding on the seeds of gentians, after which it completes development in the nests of suitable *Myrmica* ant species. The host plant and host ant species can differ at the population level within a region, and local adaptation is common, but some host switches are observed. It has been suggested that one mechanism of change is through the re-adoption of caterpillars by different ant species, either through occupation of abandoned nests or take-over of established nests by competitively superior colonies. To test this question in the lab we introduced relatively strong colonies (50 workers) of alien *Myrmica* species to the arenas of weaker colonies (two caterpillars with six workers), and to orphaned caterpillars (two caterpillars without ants). We used caterpillars from a xerophylic population of *P. alcon*, and both local hosts, *M. sabuleti* and *M. scabrinodis*, testing the possibility of host switch between these two host ant species during larval development. Most of the caterpillars were successfully readopted by alien ants, and survived well. Our results suggest higher ecological plasticity in host ant usage of this butterfly than generally thought.

## 1. Introduction

Different forms of social parasitism are well known to affect all insect taxa that live socially, where the social parasites exploit the host’s parental efforts [1]. For example, many social parasites from a wide variety of insect orders, and even different phyla, are known to exploit ants [2]. *Myrmica* ants (Hymenoptera: Formicidae) host especially diverse forms of social parasite [3]. The threatened *Phengaris* (=*Maculinea*; Lepidoptera: Lycaenidae) butterflies are probably the most intensively studied social parasites of *Myrmica* ants [4]. The caterpillars of these butterflies start their development feeding on the developing seeds of specific host plants. In the final-instar the caterpillars leave the initial food plant and mimic the odour [5,6,7,8] and the sound [9] of certain *Myrmica* species, so as to be “adopted” and raised by the ants [10]. Most *Phengaris* caterpillars are basically predators of the ant’s brood, but the Alcon Blue butterfly (*Phengaris alcon* [Denis & Schiffermüller], 1775) has a specialised “cuckoo” strategy, where the caterpillars are mostly fed by the *Myrmica* workers by trophallaxis [11]. This means that after adoption by a suitable ant colony caterpillars of *P. alcon* achieve a high social status (i.e., workers prefer them to their own larvae, even sexual brood [12]).

Initial work by Thomas et al. [10] suggested that each European *Phengaris* butterfly depends primarily on a single host ant species, as not all of the available *Myrmica* species nurse the caterpillars of a given population. Now, however, it is clear that this host ant specificity is not so strict, and these butterflies have several primary host ant species. *Phengaris* populations usually have only one or a few ‘primary host’ ant species, but these can be different among different populations of the same *Phengaris* species [4]. There is generally a geographic pattern in host use where the peripheral populations typically use a single host ant, but in Central Europe multiple host ant usage within a single population is not uncommon [4].

There are several hypotheses for apparent multiple host ant usage within the same *Phengaris* population [13], i.e., (1) ‘non host’ *Myrmica* species can tolerate ill-adapted caterpillars under benign conditions, (2) there can be generalist caterpillars which are adapted to mimic more than one host ant, (3) there can be polymorphic caterpillars within the same population that mimic different host species, (4) there can be sympatric populations exploiting different ants within the same site, and (5) artefacts, such as ant misidentification or early sampling just after the ‘adoption’ of the caterpillars, can lead to the appearance of multiple host use. Another such artefact can be when a *Myrmica* colony dies or moves to a new nest and leaves *Phengaris* caterpillars or pupae in the old nest, and another *Myrmica* species occupies the vacant nest [14,15]. Since nesting sites may be limiting for *Myrmica* ants, it is also likely that more competitive *Myrmica* colonies may invade the nest of a *Myrmica* colony nursing *Phengaris* caterpillars, displacing the original host. Although this was considered by Thomas et al. [13] as a sampling artefact, if the species that takes over the nest with caterpillars then continues to rear them through to successful pupation and emergence, this can represent genuine multiple host use, and could potentially select for generalism or polymorphism. Our aim in this study was to test the possibility of the re-adoption of “orphaned” and “invaded” caterpillars by alien *Myrmica* species. This knowledge could help to answer which of these five hypotheses is more likely (although they are not mutually exclusive).

## 2. Materials and Methods

### 2.1. Study Species, Population and Site

**Figure 1:**
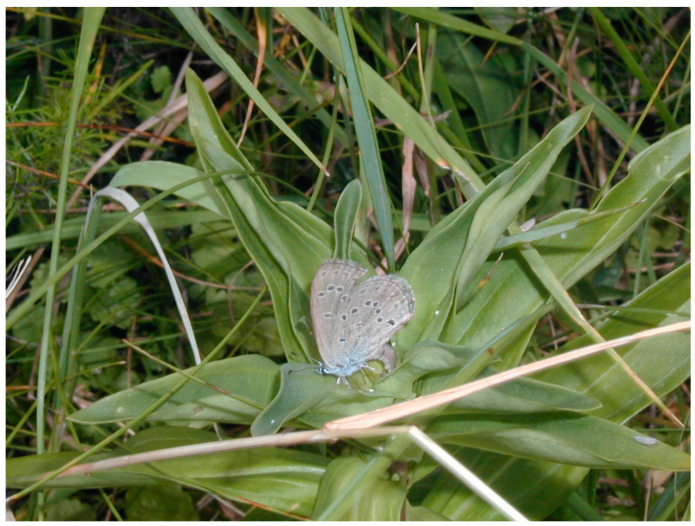
Egg laying *Phengaris alcon* female on *Gentiana cruciata*. The white dots are eggs already laid on the plant.

We chose *P. alcon* (Figure 1) as a model organism because its highly specialised “cuckoo” host ant exploitation strategy [11] is thought to be most likely to lead to local specialisation on host chemical profiles [6,11]. Another reason for choosing this butterfly was that of all the *Phengaris* species, pre-adopted caterpillars of *P. alcon* can most easily be collected in high numbers [14].

The study population belongs to the xerophilic ecotype of *P. alcon* [16]. This population inhabits a small meadow at Bükkszentkereszt (Hungary, 48°04′ N, 20°38′ E, 563 m above sea level; see [17] for a detailed description and history of this site), uses *Gentiana cruciata* L. as host plant and both *Myrmica sabuleti* Meinert, 1861 and *M. scabrinodis* Nylander, 1846 as host ants [4,17,18].

### 2.2. Collecting and Culturing

**Figure 2:**
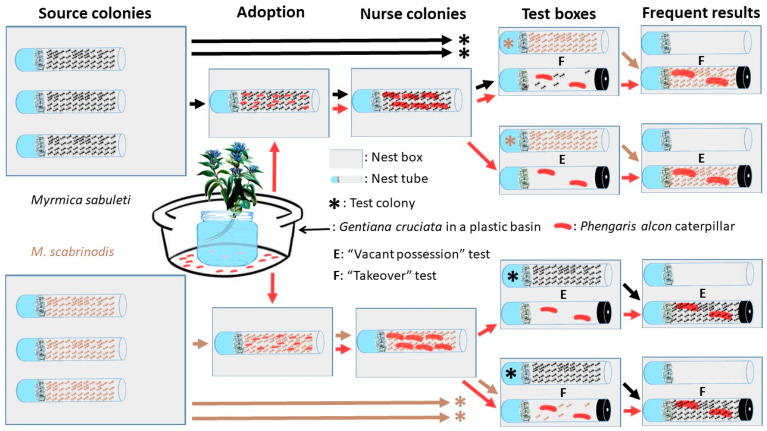
Graphical summary of the experimental set-up (see Section 2.2 and Section 2.3 for details).

Because *M. sabuleti* and *M. scabrinodis* are both polygynous [19], it is easy to collect colony fragments (“source colonies” hereafter; Figure 2) with a few (but not all) queens, hundreds of workers, and brood, without extirpating the mother colonies. Altogether 12 *M. sabuleti* and 14 *M. scabrinodis* (det. by A.T. using a Leica MZ125 10-160× magnification microscope, according to the key of Radchenko and Elmes [19]) source colonies were collected (Table 1) from meadows inhabited by *P. alcon* at Bükkszentkereszt and at the nearby Kecskeláb-rét [17] on the 15th and 16th of July 2019. The ants were collected from nests more than 10 m distance from *G. cruciata* plants to avoid potential disturbance of the *P. alcon* population and to work with, presumably, naïve colonies which have not met with *P. alcon* caterpillars before. No *P. alcon* were found within the collected nests. Source colonies contained at least 150 workers, at least one queen and brood. They were kept in plastic boxes (length: 19 cm; width: 16 cm; height: 16 cm) treated with Fluon^®^ on their inner walls to prevent ants from escaping. The floor of each box was covered with ca. 2 cm deep layer of soil from the original nest material, and the ants were provided with test tubes (length 10 cm; diameter 1 cm) within the boxes for nesting, which were part-filled with water and plugged with cotton wool (“nest tube” hereafter; Figure 2). The ants were kept at room temperature (23 °C ± 1 °C) under a natural light cycle and were fed with frozen crickets three times a week and with honey-sugar (20–20%) water solution ad libitum.

To obtain pre-adopted *P. alcon* caterpillars, 15 stems of *G. cruciata* bearing eggs of *P. alcon* (Figure 1) were collected from strong plants in the field, and moved to the lab on the 17th July. In the lab, the stems were kept in a glass of water placed in a plastic basin (Figure 2), and could be kept fresh for 2–3 weeks while the caterpillars emerged. Between 17th and 31st of July, the fourth instar caterpillars emerging from the plants were collected using a fine brush as they dropped from the flowers to the basin, and were transferred immediately into the foraging arenas (i.e., onto the soil in the boxes) of “nurse” *Myrmica* colonies, where they were rapidly adopted by the ants. These nurse colonies (Table 1, Figure 2) were derived from the source colonies, one from each, and contained 50 workers selected by emptying all nest tubes into an arena, which was then shaken to distribute the workers, queens and brood, and collecting 50 workers haphazardly with an aspirator, to provide, as far as possible, a random sample of workers from every part of the nest. No queen(s) or brood were provided to the nurse colonies to standardize the experimental nests, since there were natural differences among the source in queen and brood availability, and to avoid potential differences in caterpillar treatment due to variation in queen fertility and larval castes [12,20]. They were kept under the same conditions as source colonies but in smaller boxes (length: 16.5 cm; width: 11.5 cm; height: 6 cm) and without soil. The survival of the caterpillars was checked daily during the adoption period (between 17th and 31st of July) and in cases where we saw only a few caterpillars in a nurse colony, additional freshly dropped caterpillars were introduced. Our aim was to have a minimum of four caterpillars per nest for the different tests. This meant that we introduced 12–20 caterpillars to the nurse colonies (Table 1). The nurse colonies with caterpillars were cultured in the same way as before.

To produce “test colonies”, we separated 50 workers, without queen or brood from the source colonies between 2nd and 4th of October. Two or four test colonies were derived from each nurse colony, to be used in two tests (Section 2.3.1 and Section 2.3.2 below, Figure 2, Table A1). Test colonies were reared under the same conditions as nurse colonies.

### 2.3. Tests of Colony Take-Over

At least one month was allowed after caterpillar adoption to ensure integration into the nurse colonies and to allow some caterpillar development (see above). This length of time is sufficient to allow caterpillars from this population to develop well in the laboratory [21]. After this introductory period, in early October, we had 4–13 well developed caterpillars in each nurse colony, except for one nurse colony (B6) which did not have any surviving caterpillars (Table 1). The caterpillars were all approximately the same size (c.a. 1 cm in length), as no two-year-developing caterpillars [22] are known from this population (A.T. personal observation).

Two different tests of colony take-over were then conducted, to test different scenarios that have been proposed for host change in the field [13].

#### 2.3.1. “Vacant Possession” Test

We moved two caterpillars without ants (“orphaned caterpillars” hereafter) from each nurse colony to a new nest tube on 8th October, and these nest tubes were placed into boxes (“test box” hereafter; Figure 2) which were the same size as those used for nurse colonies. These nest tubes were fitted with polythene plugs to prevent the caterpillars leaving, but a small (2 mm^2^) passage was bored into the plugs to allow ant workers to enter and exit. After a two day acclimatization period for the orphaned caterpillars, a second nest tube containing 50 worker ants from a randomly selected heterospecific source colony (“foreign ant” hereafter) was introduced to the box on 10th October. The nest tubes of the foreign ants were not plugged (Figure 2), to encourage the ants to move to the tubes of the caterpillars with the smaller entrances (c.f. [23]). Each nurse colony with surviving caterpillars was used at least once, and if more than seven caterpillars were present, a second replicate was set up with a second, randomly chosen foreign ant colony that also had more than seven caterpillars. The test boxes were then maintained under the same conditions as the nurse colonies.

Between 10th of October and 13th of November, all nest tubes in the test boxes were checked every Monday, Wednesday and Friday for the presence of the 2 caterpillars and ants. As both *M. sabuleti* and *M. scabrinodis* start showing over-wintering behaviour at about the end of October [24,25], the caterpillars were exposed to the ants and vice versa for about 20 days from the start of the test, and had around two extra weeks at room temperature compared to the wild. A total of 40 colony pairs were established (Table A1). This test was set up to mimic the scenario in which a *Myrmica* colony moves out of a nest, leaving its *P. alcon* caterpillars behind, and the nest is then taken over by a different *Myrmica* species. For the ants, there were two possible outcomes of this test, the foreign ants either did or did not move into the tube with the caterpillars.

#### 2.3.2. “Takeover” Test

This test was set up in exactly the same way as the “Vacant possession” test, except that six workers from the original nurse colony were introduced along with the caterpillars to the partially-occluded tube in the test boxes (Figure 2). Exactly the same pairings of colonies were used for the “Takeover” and “Vacant possession” tests (Table A1). This test was set up to mimic the scenario in which a *Myrmica* colony takes over a nest from another species of *Myrmica* with a smaller colony, and in the process also takes over its *P. alcon* caterpillars. For the ants, there were three possible outcomes of this test, the foreign ants could move into the tube with the caterpillars, the nurse ants could remain with the caterpillars, or the caterpillars could be left untended.

Generally the two *Myrmica* species were easily differentiated from each other within the same box (*M. scabrinodis* is smaller than *M. sabuleti*, they have a slightly different color pattern, etc.). Furthermore, we used ant number as a tool for identification. When there were maximum 6 ants in the plugged tubes, looking different from the many foreign ants in the unplugged tubes, these few ants were identified as nurse ants. If the situation was not so clear, the inhabitants of the tubes were identified under a binocular microscope (see Section 2.2). During the first surveys it sometimes happened that a few more than 6 workers were present in the plugged tubes, and some fighting was often visible there. Such data were categorized as the caterpillars already being with the foreign host.

### 2.4. Data Analysis

All analyses were carried out in JMP v. 14 (©SAS Institute, 2019). The site from which colonies and caterpillars were collected was not included in the analysis, since this was largely confounded with the *Myrmica* species. The analyses were also repeated using site instead of foreign *Myrmica* species, with similar results, but with lower *R*^2^ values (results not shown), and investigation of differences in outcome and survivorship within sites showed the same patterns as when both sites were combined (albeit with much reduced sample sizes and correspondingly higher *p*-values; results not shown).

#### 2.4.1. Ant Outcomes

The relative proportion of the different possible outcomes of each test for the ants were analyzed separately. In cases where both caterpillars died before the end of the experiment, the distribution of ants in the last observation before the death of the last caterpillar was taken as the ant outcome. For the “Vacant possession” test, a Generalized Linear Model (GLZ) with binomial errors was used. For the “Takeover” test a multinomial logistic model was carried out. In both cases foreign ant species was included as an explanatory variable.

#### 2.4.2. Caterpillar Survivorship

In all cases where caterpillars were left unattended, they rapidly died, so this “ant outcome” was excluded from further analysis. Survivorship of caterpillars introduced to nurse colonies until the start of the experiments was compared between species and sites (and their interaction) using a GLZ with binomial errors. The proportion of tended caterpillars introduced to test colonies that were still alive at the end of the experiment was examined using a GLZ with binomial errors, with foreign ant species, test (“Vacant possession” or “Takeover”) and their interaction as main effects, and with “Ant outcome” and its interaction with foreign ant species (nested within test—this was only relevant for the “Takeover” test) as covariates. The survival of caterpillars in the experimental colonies was also compared with that of the caterpillars that remained in the nurse colonies over the same period, by including this as a third level of “test” in a second analysis. Proportional-hazards based survival analysis was also carried out, examining patterns of survivorship across the entire 20 day period, but results were similar to those provided by the GLZ analysis, showing that survivorship to the end of the experiment was representative of the whole period, but they are not presented here, as they had lower statistical power.

## 3. Results

### 3.1. Ant Outcomes

**Figure 3:**
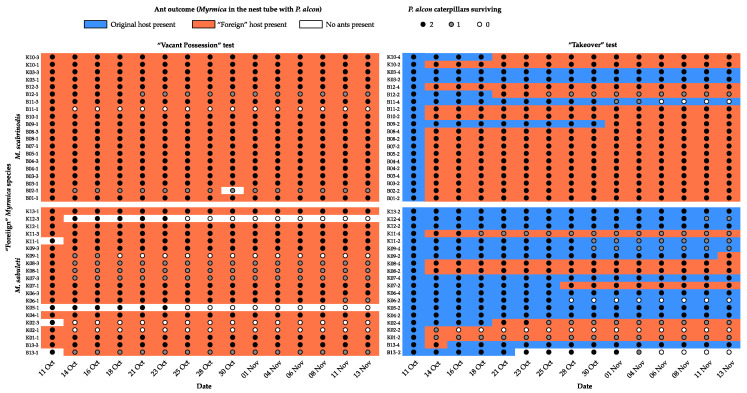
Time course of the two experiments showing which ant species occupied the tube containing the *P. alcon* caterpillars, and how many of the original 2 caterpillars were still alive at each survey.

In the “Vacant possession” test, the foreign ant workers moved in to the nest tubes with *P. alcon* caterpillars in 38 out of 40 (95%) trials (Figure 3), in general immediately after introduction. The two trials in which they did not do so were both cases in which *M. sabuleti* was the foreign ant, but unsurprisingly given the rarity of this outcome, the difference between outcomes for the two *Myrmica* species failed to reach significance (Likelihood ratio (L-R) χ^2^ = 2.88, d.f. = 1, *p* = 0.090). In the “Takeover” test, there was only a single case of caterpillars being left unattended, also in a trial with *M. sabuleti* as the foreign ant species. In the remaining 39 trials, the foreign ants took over the nest tube with the caterpillars in 25 cases (64%), generally within the first few days after introduction (Figure 3), and left the original ants to tend the caterpillars in the remaining 14 cases (36%). However, the two *Myrmica* species differed significantly in their outcomes (L-R χ^2^ = 9.56, d.f. = 2, *p* = 0.008), with *M. scabrinodis* displacing *M. sabuleti* in 17 out of 20 (85%) trials in which they were the foreign species, while *M. sabuleti* only displaced *M. scabrinodis* in 8 out of 19 (42%) trials where it was the foreign species.

### 3.2. Caterpillar Survivorship

Survivorship of caterpillars introduced into the nurse colonies until the experiments were carried out was higher in nests of *M. sabuleti* (72.8%) than those of *M. scabrinodis* (50.5%), but not significantly so (L-R χ^2^ = 0.806, d.f. = 1, *p* = 0.369), and there was no association between survivorship and either site (L-R χ^2^ = 1.72, d.f. = 1, *p* = 0.190) or the interaction of site and ant species (L-R χ^2^ = 0.487, d.f. = 1, *p* = 0.485)

**Figure 4:**
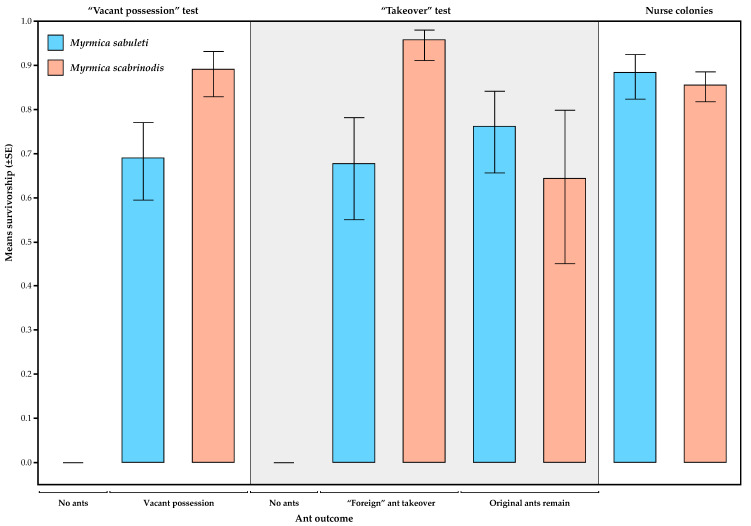
Survivorship of caterpillars of *P. alcon* over the course of the experiments under the different experimental treatments, according to “ant outcome” (see results), and in the nurse colonies over the same time period. The *Myrmica* species is that found with the caterpillars at the end of the experiment.

In the three cases where caterpillars were left unattended (see above), both caterpillars died. However, when there were ants present in the nest tube, survivorship was generally high, with 100% survival of caterpillars in 27 of 38 trials (71%) in the “Vacant possession” test and 29 of 39 trials (74%) in the “Takeover” test. The results of the GLZ comparing these tests are shown in Table 2. These show that the only significant effects are those of foreign ant species and the foreign ant × ant outcome interaction. This is because survivorship rates were generally higher when *M. scabrinodis* was the foreign ant across both tests, except for caterpillars that remained with their *M. sabuleti* nurse workers in the “Takeover” test, which had a lower survivorship (Figure 4).

Survivorship of caterpillars in the test colonies, although high, was generally lower than that of caterpillars that remained in the nurse colonies, and the same pattern was seen of higher survivorship of caterpillars when tended by *M. scabrinodis* than *M. sabuleti* (Figure 4, Table 3).

## 4. Discussion

Our results clearly show the ability of *P. alcon* caterpillars to survive for several weeks with an ant species that was not their original host. In other words, this is a likely route for host ant switching. Some of the caterpillars died during the tests, but this mortality was much lower than that experienced by caterpillars on first adoption by their primary host in our or in previous studies [5,26,27]. These results highlight the ecological plasticity of *Phengaris* caterpillars and raise new questions about their ant-mimicking strategy.

It is a well-known phenomenon that ants avoid or attack individuals from foreign colonies, whether of the same or a different species [2,28], which we have also experienced often with *Myrmica* ants in the lab [29,30]. Whether aggression takes place, and how strong it is, depends on multiple factors, including resource availability [31,32], territory or nest ownership [33,34,35], the presence of social and individual parasites [29,30], and recognition of intruders as being “non-self” [34,36], primarily through colony and species-level differences in cuticular hydrocarbons (CHCs) [37]. The level of aggression is generally directly and positively related to the dissimilarity between the CHCs of the colony and the intruders [38,39]. Initial adoption of *Phengaris* caterpillars by *Myrmica* ants also seems to be dependent on similarity in CHCs [40], with quick adoption and integration into the colony only occurring where there is a close match between CHCs of host and parasite [5]. Once integrated into the ant nest, there is further convergence in the CHC profiles of *P. alcon* caterpillars and those of their host colony workers [7], which is thought to allow the parasitic caterpillars to continue to exploit the host colony without being recognized as intruders. This means that *Phengaris* caterpillars generally mimic the CHC profiles of their host ant population, and come to resemble those of their specific host colony. One of the practical outcomes of this is that the CHC profiles of adopted caterpillars are likely to label them as intruders if encountered by workers from other *Myrmica* nests, whether of the same or different species [41].

However, it has been observed several times that caterpillars must have switched nest between species in the period between initial adoption and eclosion from the pupa. For example, Thomas and Wardlaw [14] introduced single *P. arion* (Linnaeus, 1758) caterpillars to 56 *M. sabuleti* nests in the field, but when the species of ant was identified again at eclosion of the butterflies, the ant occupying three nests was *M. scabrinodis*. During our field studies [4], we have also found *Myrmica* nests with less than 10 workers (without queen and brood) with *Phengaris* pupae or prepupal larvae (unpublished data) and also a nest which contained nine *P. alcon* pupae but no ants [15]. Laboratory studies where *P. alcon* are switched between host species, however, generally result in high mortality [7], although caterpillars are generally accepted if they are left untended by ants for a period of 4 days [7].

In our experiments we observed remarkably high levels of survival for caterpillars of *P. alcon* that switched hosts between *Myrmica scabrinodis* and *M. sabuleti* and vice versa, regardless of whether they were left untended by the previous host, or whether they were tended by a small number of workers of the previous host. Although survivorship was significantly lower than in nurse colonies, it was still generally high over the course of the experiment (Figure 4), so that integration into the experimental colonies of the new host species seems to have occurred readily. There could potentially be multiple reasons behind such acceptance. Firstly, lack of aggression may simply have been because resources available to the ants were so plentiful that the costs of discrimination and aggression may have been lower than the benefits of doing nothing. This has been observed previously in colonies of *Myrmica* ants with *P. alcon* caterpillars present [7], and under benign conditions, even rearing of *P. alcon* by other ant genera, such as *Manica*, is possible [42]. However given the size of our experimental colonies, the growth of the *P. alcon* within them, and the somewhat increased mortality relative to nurse colonies, it is likely that the cost to the colonies of rearing the *P. alcon* caterpillars was considerable.

A second possibility is that the caterpillars altered their CHC secretory behavior to more closely match the CHCs of the new host species. Such a change in CHCs after enforced host switching has been observed previously [7], probably involving the suppression of synthesis of some CHCs. A third possibility is that *P. alcon* from these populations already have a “compromise” CHC profile [6], which continues to be produced, and which allows them to relatively easily integrate into the colonies of the new host. A fourth possibility, which has received much discussion (e.g., [41,43]), but still remains relatively untested, is that caterpillars of *P. alcon* produce a CHC profile that mimics a general brood signal that is recognized across multiple *Myrmica* species. Most examinations of *Myrmica* CHCs have been carried out with worker ants, but it has been shown that *Myrmica* brood maintain CHC profiles different from the workers [44], and that CHC profiles of pre-adoption caterpillars of *P. alcon* in Denmark are more similar to those of the larvae of their hosts than to those of host workers, and that the speed adoption is directly related to how similar the CHC profiles are to those of the host larvae, even across populations [5]. It is well known that ant colonies accept unfamiliar brood [2], and this seems to be particularly common in *Myrmica* sp., where mixed colonies are easily established in the laboratory [45], and have also been observed in the field (D.R.N. personal observation). Such adoption of brood, so long as they will develop into workers that will work for the host colony, will be favored by selection, and may in turn be exploited by social parasites [41].

A final possibility is that the caterpillars of *P. alcon* use some other method to integrate into the colony [46,47]. For example, the caterpillars might communicate in a different “chemical language”, in the same way as *Lomechusa* (=*Atemeles*) beetles when the adults move from nests of *Formica* ants to *Myrmica* ants for wintering and back for reproducing [48,49], or the caterpillars could produce generalized deterrents like the ant *Formicoxenus nitidulus* (Nylander, 1846) which is a social parasite of numerous *Formica* ant species [50]. However both of these last possibilities are unlikely, since they are contradicted by the well-studied chemical adaptations of *Phengaris* caterpillars to certain host ant species [5,6,7,8].

Adoption and integration of *P. alcon* caterpillars within *Myrmica* nests may also depend on the social structure of nests, particularly the presence [20] and number [29,41] of queens, and the treatment of caterpillars will also likely depend on the presence of different castes of brood [12]. Hence our simplified nests with only workers may have influenced the takeover process, but polygynous *Myrmica* species (including *M. sabuleti* and *M. scabrinodis*) are also polydomous (having several nests per colony), and many of these sub-nests may lack queens and brood [51].

Regardless of which mechanism resulted in the acceptance of *P. alcon* by new hosts in our experiments, it may be highly relevant that the two host species used are close relatives [52,53], both belonging to the “scabrinodis” group within *Myrmica* [19]. Their worker CHC profiles are therefore rather similar [54], and their larval profiles may be even more so. A parallel situation is the multiple host ant using hygrophilous *P. alcon* populations in North-Western Europe, where *M. rubra* (Linnaeus, 1758) and *M. ruginodis* Nylander, 1846 are the host ant species of the same populations [4,55]. Both *M. rubra* and *M. ruginodis* belong to the *rubra*-group [19] and are genetically closely related species [52] with a great deal of overlap their worker CHC profiles [54], and with larval CHC profiles that only differ by a single compound [5]. However, there are xerophilic populations of *P. alcon* in Central-Europe which use *M. sabuleti* and/or *M. scabrinodis* and also use *M. schencki* Viereck, 1903 [4]. The latter ant belongs to the *schencki*-group [19], is not so closely related genetically to *M. scabrinodis* and *M. sabuleti* as they are each other [52] and has therefore a rather different CHC profile from these two *Myrmica* species [54]. We have not had access to a site where we would be able to collect both *M. schencki* and also *M. sabuleti* and/or *M. scabrinodis* in high numbers, and where these ants are all used by *P. alcon* [4], but it would be interesting to repeat our experiment with such a combination.

Despite the genetic similarity of *M. scabrinodis* and *M. sabuleti* [52,53] and the similarity of their CHC profiles [54], we did find differences between these two species in our experiments. In particular, a lower proportion of caterpillars survived the period between their adoption and the starting of the test (Table 1) in *M. scabrinodis* than in *M. sabuleti* nurse colonies, although the difference was not statistically significant. Most of these caterpillars died within a day after we introduced them to the nurse colonies. On the other hand, the survival of adopted caterpillars was significantly higher with *M. scabrinodis* than *M. sabuleti* test colonies, but ultimately did not depend on the type of test (“Vacant possession”/”Takeover”; Figure 1 and Figure 2). The test type rather seemed to influence the start of mortality, which occurred earlier when no original host workers were present (Figure 3), but not significantly so. Secondly, in the “Takeover” test, there were cases where the foreign *Myrmica* species left the caterpillars with the few original nursing workers, and this outcome was significantly more frequent for *M. scabrinodis* than *M. sabuleti* (Table 2, Figure 4). Finally, we should emphasize that *M. scabrinodis* were better able to defend their nests from takeover than *M. sabuleti* (Section 3.1; Figure 3). There were 11 colonies of *M. scabrinodis* (with 2–6 workers) and three of *M. sabuleti* (with 1–5 workers) that defended their nest, and in most cases their caterpillars too, until the end of the “Takeover” tests. This means that they (1–6 workers and the caterpillars) survived well for a month within a small test box by feeding on the same food source as the 50 workers of the foreign species, which left them alive. There was also one colony (B-13-4) which drove out the foreign ants successfully from its plugged nest tube (see: Section 2.3.2), albeit only for a single survey period (Figure 3). These observations emphasize the importance of a deeper knowledge of the species-specific behavior of *Myrmica* ants, if we would like to understand the suitability of different *Myrmica* species as *Phengaris* hosts.

It is all well and good to show host-switching under the laboratory conditions that we set up, but is it likely to happen in the field? Radchenko and Elmes [19] describe the mobility and migration behaviour of *Myrmica* colonies, and Thomas and Wardlaw [14] estimated that as many as 68% of nests of *M. sabuleti* that had *P. arion* caterpillars introduced in late summer had been taken over by another colony by the following summer (14% of them by *M. scabrinodis*), which they concluded was most likely through the original host colony abandoning the nest. While *P. alcon* should have a much smaller impact on its host colonies than *P. arion* [11], it is still a virulent parasite [5], and our present results suggest that it is quite likely that *Phengaris* caterpillars/pupae are readopted by a new host ant in nature, either through the original nest being abandoned, or through takeover. Our preliminary unpublished records of pupation also support this interpretation, as overwintered caterpillars from the same populations of *P. alcon* used in this study pupated successfully when moved to foreign ant colonies (from *M. sabuleti* to *M. scabrinodis* and vice versa). In summary, our findings mostly support hypotheses 1, 2 and 5 for multiple host use, as presented in the introduction, but do not exclude the possibility of hypotheses 3 and 4.

*Phengaris* butterflies have a highly specialised life cycle, and for a long time were thought to be strictly host specific [10]. This “strict specialisation theory” resulted in the xerophilic and hygrophilic ecotypes of *P. alcon* being treated erroneously as separate species, based on their different habitat types, host plants and host ants [16]. What is more, the hygrophilic form of *P. alcon* has been suggested to be three subspecies or cryptic species based on the three different host ant species used by different populations [56]. However, the background of such a multiple host ant usage of *Phengaris* species is the coevolution of *Phengaris* butterflies and their *Myrmica* hosts [4] in a geographic mosaic [57]. It means that the different populations of *Phengaris* species are able to adapt to different host ant species, and indeed some consistently use multiple hosts [4]. This study emphasizes the likelihood of *P. alcon* caterpillars changing host ant species during their development and that the ecological plasticity of *Phengaris* butterflies is higher than initially thought.

## 5. Conclusions

Our aim was to test the possibility of re-adoption of the social parasitic *Phengaris alcon* caterpillars by a new *Myrmica* ant species. The results clearly show the ability of *P. alcon* caterpillars to change host ant through nest takeover, at least with the species and social structure of *Myrmica* ants that we used.

## Figures and Tables

**Table 1:** The number of pre-adopted *Phengaris alcon* caterpillars introduced to the *Myrmica sabuleti* and *M. scabrinodis* nurse colonies, the number of caterpillars alive at the start of the tests, and the number of caterpillars remaining in the nurse colonies after the experiments were established (i.e., after 4 or 8 caterpillars were used for the experimental set-up—see Table A1).

		Number of *P. alcon* Caterpillars		
*Myrmica* sp.	Colony ID	Introduced	Alive	Remaining
*sabuleti*	B13	18	12	4
	K1	13	10	6
	K2	14	12	4
	K4	14	13	9
	K5	13	9	5
	K6	12	9	1
	K7	12	12	4
	K8	16	9	1
	K9	15	10	2
	K11	20	10	2
	K12	13	9	1
	K13	13	11	7
	**Total**	**173**	**126**	**46**
*scabrinodis*	B1	12	7	3
	B2	13	4	0
	B3	14	8	0
	B4	14	8	0
	B5	12	4	0
	B6	15	0	0
	B7	13	5	1
	B8	12	11	3
	B9	15	7	3
	B10	14	7	3
	B11	16	8	0
	B12	16	9	1
	K3	15	9	1
	K10	13	11	3
	**Total**	**194**	**98**	**18**

**Table 2:** Results of analysis of survival over the 20 days of the tests using a generalized linear model with binomial errors. The replication unit for this analysis is the test colony (n = 40). The significance of changes in deviance associated with different terms in the model was assessed using Likelihood-ratio (L-R) χ^2^ tests. Potential sources of variation in survivorship were: Foreign ant species—The introduced *Myrmica* species (the species that was not used in nurse colonies), either *M. scabrinodis* or *M. sabuleti*. The Test (“Vacant possession” or “Takeover”), and the Ant outcome, which was nested within Test, since only a single outcome was possible for the “Vacant possession” test (takeover by the foreign ant species), while two outcomes (continued tending by the original host ant species or takeover by the foreign ant species).

Source of Variation	DF	L-R χ^2^	*p*
Foreign ant species	1	6.904	0.0086
Test	1	0.007	0.9337
Ant outcome [Nested in Test]	1	2.546	0.1106
Foreign ant species × Test	1	0.083	0.7738
Foreign ant species × Ant outcome [Nested in Test]	1	4.726	0.0297

**Table 3:** Results of analysis of survival over the 20 days of the tests plus comparison with the survival in the nurse colonies over the same period, using a generalized linear model with binomial errors. The replication unit for this analysis is the test or nurse colony (n = 60). The significance of changes in deviance associated with different terms in the model was assessed using Likelihood-ratio (L-R) χ^2^ tests. Potential sources of variation in survivorship were: *Myrmica* sp.—The introduced *Myrmica* species for the test colonies, and the nurse species for the nurse colonies, either *M. scabrinodis* or *M. sabuleti*. The Test (“Vacant possession”, “Takeover”, or nurse colony), and their interaction.

Source of Variation	DF	L-R χ^2^	*p*
*Myrmica* sp.	1	14.64	<0.0001
Test	2	6.53	0.0382
*Myrmica* sp. × Test	2	1.86	0.3951

## Appendix A

**Table A1:** Summary of the experimental nests used in the two experiments “Vacant possession” and “Takeover”. The same 40 combinations of “nurse” and “foreign” colonies were used for both experiments, with different *P. alcon* caterpillars and worker ants. The identity of the “foreign” *Myrmica* species is shown—the nurse colony was of the other species. See Table 1 for an explanation of colony IDs.

Nurse Colony		“Foreign” Colony		Experimental Nest ID	
Colony ID	Site	Colony ID	*Myrmica* Species	“Vacant Possession”	“Takeover”
B01	Bükkszentkereszt	K13	*scabrinodis*	B01-1	B01-2
B02	Bükkszentkereszt	K06	*scabrinodis*	B02-1	B02-2
B03	Bükkszentkereszt	K02	*scabrinodis*	B03-1	B03-2
B03	Bükkszentkereszt	K07	*scabrinodis*	B03-3	B03-4
B04	Bükkszentkereszt	K09	*scabrinodis*	B04-1	B04-2
B04	Bükkszentkereszt	B13	*scabrinodis*	B04-3	B04-4
B05	Bükkszentkereszt	K12	*scabrinodis*	B05-1	B05-2
B07	Bükkszentkereszt	K09	*scabrinodis*	B07-1	B07-2
B08	Bükkszentkereszt	K11	*scabrinodis*	B08-1	B08-2
B08	Bükkszentkereszt	B13	*scabrinodis*	B08-3	B08-4
B09	Bükkszentkereszt	K11	*scabrinodis*	B09-1	B09-2
B10	Bükkszentkereszt	K06	*scabrinodis*	B10-1	B10-2
B11	Bükkszentkereszt	K08	*scabrinodis*	B11-1	B11-2
B11	Bükkszentkereszt	K08	*scabrinodis*	B11-3	B11-4
B12	Bükkszentkereszt	K04	*scabrinodis*	B12-1	B12-2
B12	Bükkszentkereszt	K12	*scabrinodis*	B12-3	B12-4
B13	Bükkszentkereszt	B08	*sabuleti*	B13-1	B13-2
B13	Bükkszentkereszt	B04	*sabuleti*	B13-3	B13-4
K01	Kecskeláb-rét	K03	*sabuleti*	K01-1	K01-2
K02	Kecskeláb-rét	B03	*sabuleti*	K02-1	K02-2
K02	Kecskeláb-rét	K03	*sabuleti*	K02-3	K02-4
K03	Kecskeláb-rét	K02	*scabrinodis*	K03-1	K03-2
K03	Kecskeláb-rét	K01	*scabrinodis*	K03-3	K03-4
K04	Kecskeláb-rét	B12	*sabuleti*	K04-1	K04-2
K05	Kecskeláb-rét	K10	*sabuleti*	K05-1	K05-2
K06	Kecskeláb-rét	B02	*sabuleti*	K06-1	K06-2
K06	Kecskeláb-rét	B10	*sabuleti*	K06-3	K06-4
K07	Kecskeláb-rét	K10	*sabuleti*	K07-1	K07-2
K07	Kecskeláb-rét	B03	*sabuleti*	K07-3	K07-4
K08	Kecskeláb-rét	B11	*sabuleti*	K08-1	K08-2
K08	Kecskeláb-rét	B11	*sabuleti*	K08-3	K08-4
K09	Kecskeláb-rét	B07	*sabuleti*	K09-1	K09-2
K09	Kecskeláb-rét	B04	*sabuleti*	K09-3	K09-4
K10	Kecskeláb-rét	K05	*scabrinodis*	K10-1	K10-2
K10	Kecskeláb-rét	K07	*scabrinodis*	K10-3	K10-4
K11	Kecskeláb-rét	B08	*sabuleti*	K11-1	K11-2
K11	Kecskeláb-rét	B09	*sabuleti*	K11-3	K11-4
K12	Kecskeláb-rét	B05	*sabuleti*	K12-1	K12-2
K12	Kecskeláb-rét	B12	*sabuleti*	K12-3	K12-4
K13	Kecskeláb-rét	B01	*sabuleti*	K13-1	K13-2
